# Influence of HIV/AIDS Infection on Immunological and Nutritional Status in Adults and Older Adults: A Cross-Sectional Study in Kingdom of Bahrain

**DOI:** 10.3390/geriatrics8050088

**Published:** 2023-09-04

**Authors:** Fatima Alabdulali, Afnan Freije, Mariam Al-Mannai, Jameela Alsalman, Fatima Ahmed Buabbas, Mariangela Rondanelli, Simone Perna

**Affiliations:** 1 Department of Biology, College of Science, University of Bahrain, Sakhir Campus, Zallaq P.O. Box 32038, Bahrain; 2 Department of Mathematics, College of Science, University of Bahrain, Sakhir Campus, Zallaq P.O. Box 32038, Bahrain; 3 Al Salmaniya Medical Complex, Ministry of Health, Manama 435, Bahrain; 4 IRCCS Mondino Foundation, 27100 Pavia, Italy; 5 Department of Public Health, Experimental and Forensic Medicine, University of Pavia, 27100 Pavia, Italy; 6 Department of Food, Environmental and Nutritional Sciences, Division of Human Nutrition, Università Degli, Studi di Milano, 20133 Milan, Italy

**Keywords:** HIV/AIDS patients, nutritional, biochemical, markers, immunological disease

## Abstract

*Background and Objectives*: HIV infection is a global public health problem that can lead to the progression of AIDS. Nutritional status and biochemical markers can significantly contribute to the progression of AIDS in HIV/AIDS patients. The main objective of this study is to examine the association between nutritional and biochemical markers as well as BMI in HIV/AIDS patients in the kingdom of Bahrain. *Methods*: A retrospective cohort study, including 300 patients (248 males and 52 females) with HIV/AIDS in Bahrain, was carried out. Various biochemical markers were collected from patients’ medical records, including CD$4^{+}$ T cell count, albumin, Hb, HCT, MCV, WBCs, and creatinine. A semi-structured questionnaire using a standardized food frequency questionnaire (FFQ) was used, from which total energy and total macronutrients were calculated. *Results*: The mean BMI of the participants was 27.20 kg/m^2^, and none of the participants had a BMI lower than 18.5 kg/m^2^ (underweight). The majority of patients’ dietary intake of macronutrients and total calorie intake were either within or above the recommended RDA levels. The results also showed that all of the mean values of the nutritional and biochemical markers (CD$4^{+}$ T cell count, albumin, Hb, HCT, MCV, WBCs, and creatinine) were within the normal reference ranges. A significant positive correlation between CD$4^{+}$ T cell count, Hb, HCT, and albumin at the <0.05 level was found. There was no significant correlation between CD$4^{+}$ T cell count and MCV, WBCs, and creatinine. A positive significant correlation was found between BMI, CD$4^{+}$ T cell count, and WBCs at the <0.01 level. *Conclusion*: The BMI values were significantly correlated with the biochemical markers of AIDS progression. The dietary patterns of the participants were undiversified, with a high prevalence of obesity and overweight. Malnutrition among this study population was not present.

## 1. Introduction

The association between nutritional status, disease stage, and prolonged degradation of the immune system emphasizes that nutrition can significantly contribute to the progression of acquired immunodeficiency syndrome (AIDS). Along with human immunodeficiency virus (HIV) infection and compromised immunity, there are a number of risk factors, including muscle wasting, fat mass loss, reduced appetite, gastrointestinal problems, and a hypermetabolic state. HIV weakens and obliterates the immune system, which lowers the body’s ability to fight off infections and increases mortality rate [1].

The correlation between nutritional status and the progression of AIDS is a complex area of study, yet it has been shown that poor nutrition may place additional stress on an already weakened immune system. Poor nutritional status, especially malnutrition, places higher energy demands on patients and their immune systems, leading to a number of risk factors, including opportunistic infections, malignancies, debilitation, and death [2]. The difficulty in maintaining a nutritious diet as AIDS progresses has been linked to biological and social factors that play huge rule in diminishing the individual’s ability to consume food. Consequently, an adequate nutrition and care plan for a population living with HIV/AIDS should always be given priority [3,4].

One of the circumstances responsible for poor nutritional status in an HIV-infected person is the reduction in patients’ appetite due to difficulty ingesting food, opportunistic infections, illness, or even psychological compromises. In addition, poor nutritional status can be commonly associated with the adverse effects of the medications used to treat AIDS patients or any related illness, which cause nausea and vomiting. At the same time, frequent bacterial infections accompanied by diarrhea can lead to poor absorption of nutrients [5,6,7,8]. In addition, HIV causes severe damage to the gastrointestinal tract by causing flattening of the villi of the small intestine cells. Eventually, this leads to several malabsorption processes regarding carbohydrate and fat, thereby compromising the absorption of fat-soluble vitamins such as vitamins A and E.

These vitamins are essential for the immune system to function properly and are required during illness and infections. Iron deficiency also very commonly causes fatigue among patients, which further decreases their consumption of food and nutrient absorption. Therefore, malnutrition is considered a sign of disease progression among HIV-infected patients [5,6,7,8]. An altered metabolism has also been shown to be prevalent in HIV-infected individuals; each patient presents with varying degrees of alteration in their metabolic processes. These metabolic changes affect their nutritional requirements and consumption. Thus, analyzing changes in the concentrations of some biochemical markers linked to nutritional status among HIV-infected people contributes to the early detection of malnutrition [9,10,11,12,13,14,15]. Additionally, dyslipidemia develops because of the complex metabolic changes that occur frequently during infection. The dyslipidemia associated with HIV infection is exemplified by reduced levels of high-density lipoprotein cholesterol (HDL-C), elevated total cholesterol (TC), elevated low-density lipoprotein cholesterol (LDL-C), and high triacylglycerol (TGC) levels, as well as a significant decrease in albumin levels. Simultaneously, malnutrition is associated with a deficiency in nutritional intake and immunological status, presenting as low body mass index (BMI), anemia, dyslipidemia, and hypoalbuminemia [9,10,11,12,13,14,15].

HIV infection is a global public health burden with a high prevalence rate in locations where malnutrition is also a major concern. The idea of improving food access for undernourished people, regardless of their disease status, is not new, yet fundamental issues remain regarding the most effective strategies to incorporate nutritional interventions into HIV care plans. The distinction between food and nutrition as well as the idea that the amount of food is not synonymous with nutritional content must be highlighted. This has received less attention because of the urgency of the situation and the understandable instinct to get whatever food is available to those who are in need. The detrimental impacts of under-nutrition, insufficient food consumption, and HIV infection necessitate concentrated efforts to develop and execute efficient cross-sectoral solutions [16].

Early nutritional intervention can preserve body mass in individuals living with HIV who have signs of active weight loss. Malnutrition is one of the earlier complications of HIV infection to be detected, which results in waste syndrome. It should be classified as a prognostic factor in advanced HIV stages; thus, malnutrition is widely considered an HIV-associated complication. 

Several clinical trials have been carried out to evaluate the effectiveness of supplementation with required nutrients at different stages of HIV infection as well as combining formula foods for treating malnutrition, along with medications. Therefore, enhanced nutritional status may guarantee better health in HIV-infected individuals [2]. AIDS negatively compromises individuals’ ability to consume, utilize, and acquire food, leading to a range of risk factors from poor nutritional status to weight loss and even morbidity. Patients with a body mass index (BMI) lower than 18.5 kg/m^2^ have a higher level of AIDS progression. In addition, as AIDS progresses, several changes in biochemical markers can be detected, which are considered common issues resulting from HIV infection [17] (Hailemariam et al., 2013).

As shown by Nyamweya et al., there were no differences in biomarker levels at the same stage of disease progression in HIV-1 and HIV-2 infections. In particular, biomarkers, β2-m, neopterin, and sUPAR were significantly elevated 6–8 years before patients died compared with the patients who survived [18].

The use of antiretroviral therapy (ART) can have an adverse impact on these disorders. The biochemical markers that can be affected are cluster of differentiated CD4^+^ T helper cells (CD$4^{+}$ T cells), albumin, hemoglobin (Hb), hematocrit (HTC), mean corpuscular volume (MCV), white blood cells (WBCs), and creatinine. The value of these biochemical markers can be lower than normal as AIDS progresses. Thus, the early assessment of nutritional status and functional biochemical markers in routine patient checkups is crucial to delaying the progression of the disease [19].

In Bahrain, the association between nutritional and chemical biomarkers and the progression of AIDS among patients has not yet been studied. The main objectives of this study were to examine the association between nutritional and biochemical markers that contribute to AIDS progression, and to analyze the nutritional factors that could increase/decrease the progression of AIDS in Bahraini citizens.

## 2. Materials and Methods

### 2.1. The Study Population

This study is a retrospective cohort study conducted during the period of August 2021 to October 2021. 

All individuals with HIV/AIDS who had a medical record at Salmaniya Medical Complex (SMC) (the only HIV/AIDS center in the Kingdom of Bahrain) were included in the study. The study population included 308 individuals (257 males and 51 females).

Data obtained from the medical records, including the latest results of the patients’ biochemical markers, such as CD$4^{+}$ T cell count, albumin, Hb, HCT, MCV, WBCs, and creatinine, were supplied by SMC.

### 2.2. Ethical Approval

Permission to conduct this study was obtained from the Deanship of Graduate Studies and Scientific Research at the University of Bahrain (UOB) on 6 April 2021 (Decision No. 2021/77). Approval was also obtained from the Secondary Health Care Research Committee (SHCRC) on 14 June 2021 (Decision No. 83140621). Verbal consent from participants was obtained before conducting the phone interviews due to COVID-19.

### 2.3. Food Frequency Questionnaire

A semi-structured questionnaire that consisted of the standardized food frequency questionnaire (FFQ) from the Harvard Food Frequency Questionnaire Form was completed via phone interviews. However, only 81 patients agreed to fill in the FFQ.

The questionnaire consisted of two parts. The first part included demographic data such as age, gender, occupation, educational level, weight in (kg) and height in (cm). The second part included the frequency of the consumption of foods in different groups. 

Participants were asked to indicate how often on average they had eaten the specified amount of each food during the past year. The were given the choices of never or less than once per month, 1–3 times per month, once per week, 2–4 times per week, 5–6 times per week, once per day, 2–3 times per day, 4–6 times per day, and 6+ times per day. The FFQ of each patient was calculated to estimate average intake of calories and macronutrients as well as stratified to obtain a list of daily consumed food.

### 2.4. Estimation of Macronutrient Intake

Patients’ dietary habits were assessed to obtain the frequency and portion size of food and beverages consumed over the past year. Patients’ average calories and macronutrient intake, including carbohydrate, fat, and protein, were calculated using the method of Willett (2012) [20].

Each frequency value was multiplied by the food’s calorie, carbohydrate, fat, and protein content using the method of Willett (2013) [20]. 

Based on patients’ choices in the FFQ, the sum of each table gave us the average intake of calories, grams of carbohydrate, grams of fat, and grams of protein. In addition, participants’ responses in the FFQ were analyzed and foods that were frequently consumed on a daily basis were recorded by count. This will give us an outlook on the most consumed foods among patients. The information regarding the calories and macronutrient contents of foods was obtained from the Diet Controller application version 2.1.0 [21].

The total calories and macronutrient content of the diets were calculated based on the World Health Organization (WHO) recommendations for HIV-infected individuals [22]. The WHO recommendations are a 10% increase in calories above the general recommendation for healthy adults [23], and a 12–15% increase for protein. However, there is no recommendation to increase carbohydrate and fat above the general recommendation for healthy adults. Therefore, it was recommended that HIV-infected individuals should consume 45–65% of their total calories from carbohydrates and 20–35% of their total calories from fat [22,23].

### 2.5. Anthropometric Measurements

Patients’ weight and height were used for the calculation of body BMI, defined as weight in kilograms divided by the square of height in meters (kg/m^2^) [24]. BMI was stratified according to the WHO criteria: <17 (moderate-to-severe malnutrition), 17 to <18.5 (mild malnutrition), >18.5 to 25 (normal nutrition), and >25 kg/m^2^ (overweight and obese) [18].

### 2.6. Statistical Analysis

Data were analyzed using the Statistical Package for Social Sciences (SPSS version 28). Descriptive statistics were presented as means ± standard deviations (SD), ranges (minimum and maximum), and percentiles. Normal distribution of data was assessed using an independent samples t-test as well as a histogram graph. Two-tailed Pearson correlation was used to correlate numeric variables such weight, height, CD$4^{+}$ T cell count, albumin, Hb, HCT, MCV, WBCs, and creatinine levels between HIV-infected patients. Binary logistic regression and one-way ANOVA were used with CD$4^{+}$ T cell count as the dependent variable to correlate its value with the other variables.

Statistical analyses were conducted taking into account patients’ current treatment status, and CD4^+^ count was used as a marker for overall HIV/AIDS status/progression.

Descriptive statistics followed by chi-square selection were used for the generation of the odds ratio and confidence interval. *p*-values of less than 0.05 were considered statistically significant.

## 3. Results

The demographic characteristics of the participants are shown in Table 1. Males compromised 80.51% of the participants, and 16.88% were female. The age range of participants was 60–85 years old. The levels of education were primary education (17.28%), intermediate education (16.04%), secondary education (39.50%), bachelor’s degree (B.Sc.) (25.92%), and higher education (1.23%). 

Based on the information obtained from the analysis of the FFQ, 73 of the participants reported that they consumed white bread daily, while 64 participants consumed white rice as their main meal for lunch every day. In addition, 15 participants stated that they consumed potatoes in the form of French fries daily. None of the participants stated that they consumed higher-fiber forms of carbohydrate daily such as oatmeal, wholegrain bread, or brown rice. Meanwhile, the counts of high-protein foods that participants reported consuming daily were eggs with 45 counts, followed by yogurt with 31 counts and beans with only 6 counts. Tea was among the most-consumed caffeinated drinks, with 75 participants reporting that they consumed tea daily, followed by coffee with 56 counts, and the least consumed, soft drinks, with only 5 counts. The general consumption of fruits and vegetables was moderate. Among vegetables, arugula (rocket), onions, and tomatoes were the most consumed, and both apples and oranges were among the most-consumed fruits (Table 2).

As shown in Table 3, the dietary assessment of 81 participants was used to calculate the nutrient intake levels including caloric intake, carbohydrate, protein, and fat. Of the participants assessed, 32.09% of males and 6.17% of females had inadequate caloric intake compared to the recommended dietary allowance (RDA) values. The mean caloric intake value was 2232 ± 509.96 Kcal/day for males and 2290 ± 425.46 Kcal/day for females.

As for protein, 2.47% of males and 6.17% of females were below the RDA recommendation, with a mean intake of 66.76 ± 36.35 g/day for males and 65.35 ± 11.70 g/day for females. Regarding lipids, the mean intake was 36.36 ± 10.92 g/day for males and 37.04 ± 7.74 g/day for females. In addition, 9.87% of males and 8.64% of females were below the RDA recommendation in terms of lipid intake. Carbohydrate intake among participants showed a mean intake of 364.04 ± 73.46 g/day for males and 333.18 ± 87.68 g/day for females. Carbohydrate consumption was above the RDA recommendation among all male participants (70.37%) as well as all female participants (29.63%).

The description and classification of BMI are illustrated in Table 4. In total, 38.27% had a normal weight, 37.03% of participants were overweight, 18.51% of participants were obese class I, 4.93% of participants were obese class II, and 1.23% of participants were obese class III. A mean BMI value of 27.20 kg/m^2^ was recorded.

**Figure 1:**
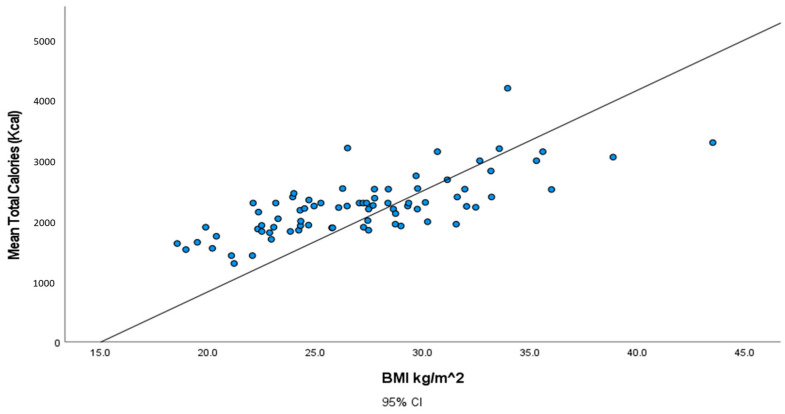
Illustration of the association between total calories (Kcal) and body mass index (BMI) (kg/m^2^).

The correlation between calorie intake and individuals’ BMI is illustrated in Figure 1. The plot reveals a positive correlation in which individuals with the highest calorie intake have a higher value of BMI.

Table 5 displays the correlation coefficients between the BMI, total calories, total carbohydrates, total lipids, and total proteins consumed. All of the correlations are significant at *p*-values ˂ 0.01 level since all of the *p*-values are less than 0.0001.

The mean CD$4^{+}$ T cell count, albumin, Hb, HCT, MCV, WBCs, and creatinine were found to be 620.91 ± 673.40 cells/${\mathrm{mm}}^{3}$, 42.77 ± 5.62 g/L, 13.51 ± 2.31 g/L, 42.15 ± 7.03%, 83.13 ± 10.28 g/L, 6.16 ± 2.59 $\times 10^{9}$/L, and 84.19 ± 57.28 mg/dL, respectively (Table 6). The results show that all of the mean values are within the normal reference ranges [26,27].

**Figure 2:**
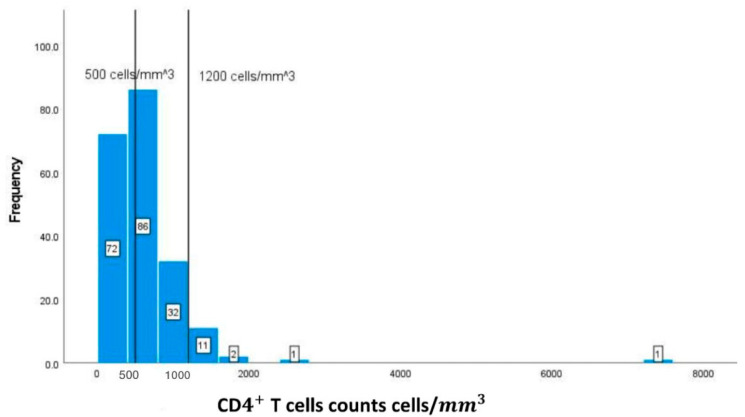
Classification frequencies (histograms) of CD$4^{+}$ T cell counts among patients.

**Figure 3:**
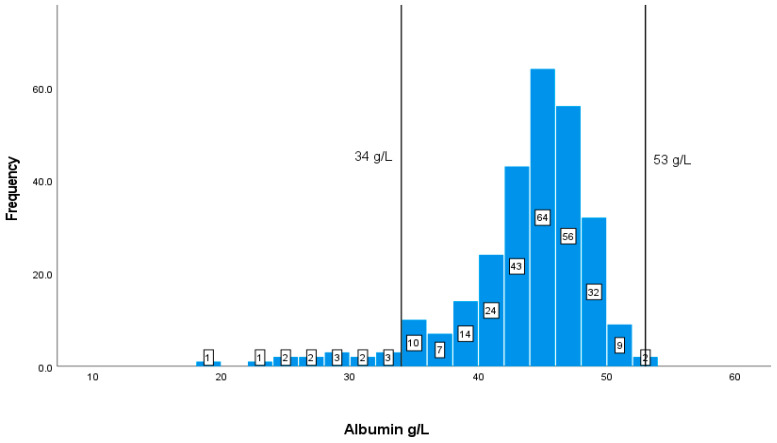
Classification frequencies of albumin among patients.

**Figure 4:**
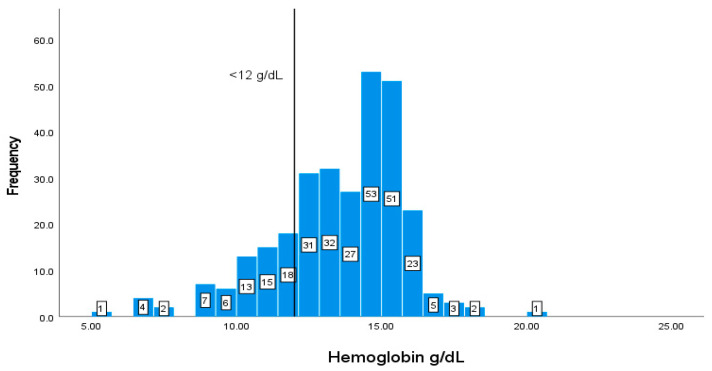
Classification frequencies of hemoglobin among patients.

**Figure 5:**
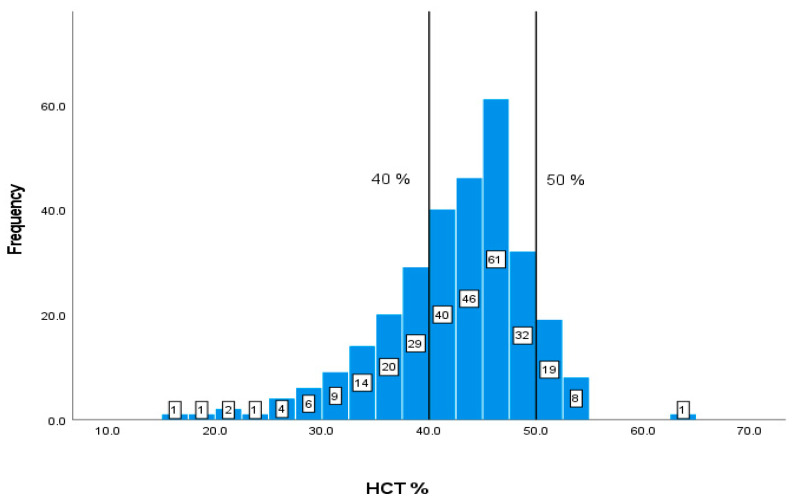
Classification frequencies (histograms) of HCT% among patients.

**Figure 6:**
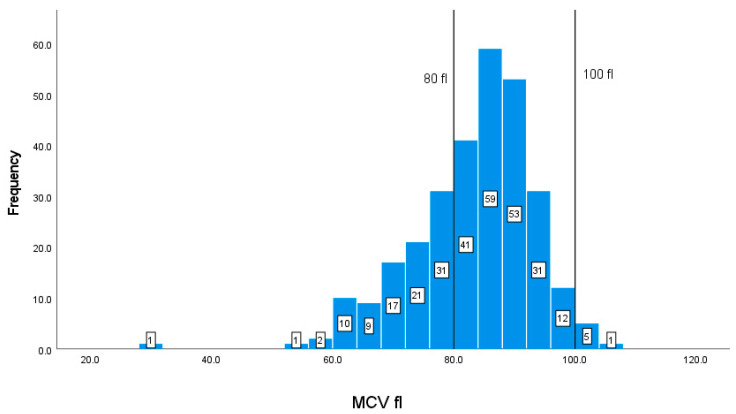
Classification frequencies (histograms) of MCV among patients.

**Figure 7:**
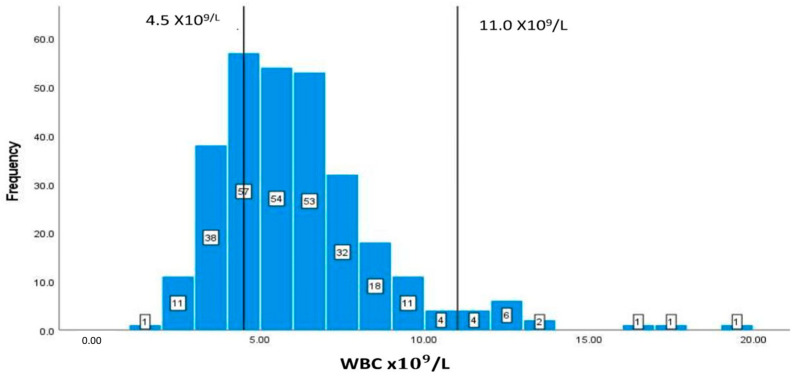
Classification frequencies (histograms) of WBCs among patients.

**Figure 8:**
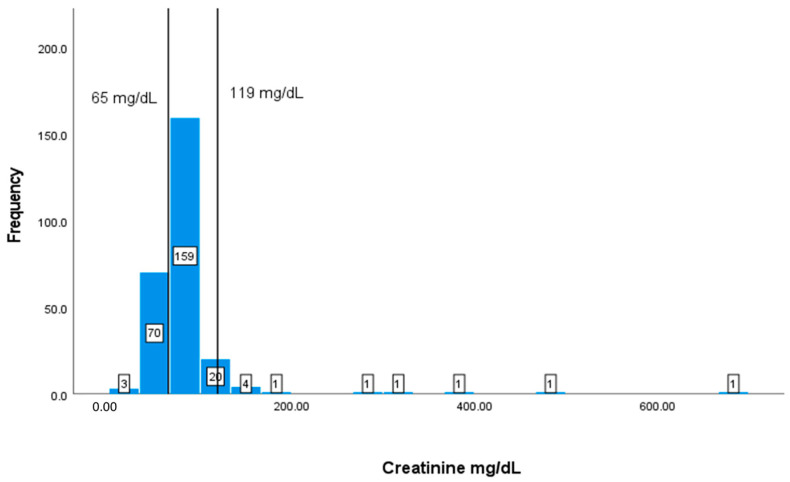
Classification frequencies (histograms) of creatinine among patients.

The values and frequencies of the patients’ biochemical markers, including CD$4^{+}$ T cell count, albumin, Hb, HCT, MCV, WBCs, and creatinine, are illustrated in Figure 2, Figure 3, Figure 4, Figure 5, Figure 6, Figure 7 and Figure 8. The values of each biochemical marker were stratified and illustrated as histograms to enable us to look at their distribution in comparison to the normal range. Additionally, two vertical lines in each histogram were added to indicate the normal range limit; all values out of their ranges are considered abnormal.

In Figure 2, the normal level of CD$4^{+}$ T cells ranges from 500 cells/${\mathrm{mm}}^{3}$ to 1200 cells/${\mathrm{mm}}^{3}$. The graph represents a right-skewed distribution of the data, meaning that the mean is to the right of the median. In addition, 94 (45.85%) out of the 205 available values for CD$4^{+}$ T cell count have an CD$4^{+}$ T cell count below 500 cells/${\mathrm{mm}}^{3}$.

Albumin shows a normal distribution of data. Most of the albumin readings for patients are within the normal range of 35–53 g/L. The available measures of albumin are 275, of which only 14 patients are below the normal level, and none have elevated levels, according to the graph (Figure 3).

Values of Hb of less than 12 g/dL represent values that are below the recommendation. Most of the patients have normal levels of Hb, in which 61 (20.74%) patients out of 294 have values of less than <12 g/dL (Figure 4).

A total of 87 (29.59%) out of 294 patients have values of HCT% lower than 40% and 28 patients have elevated values of more than 50% (Figure 5).

The normal MCV range is 80–100 fl, as shown by the vertical lines on the graph. Out of the 294 patients with available blood data, there are 92 (31.29%) patients with values of less than 80 fl, and only 6 with elevated levels of more than 100 fl (Figure 6).

Table 7 displays the correlation coefficients between all of the nutritional and biochemical markers. It can be seen from the table that there is a significant correlation of albumin with Hb, HCT, and CD4^+^ T cells (*p* ˂ 0.01). There is also a significant correlation between HCT, Hb, and MCV (*p* ˂ 0.01).

The linear regression model using the nutritional and biochemical markers was fitted against the CD4^+^ T cells (Table 8). It was found that only albumin has a significant coefficient.

The normal WBC range is 4.5–11 × $10^{9}$/L, as shown by the vertical lines in Figure 7. The data of this marker represent a right-skewed distribution similar to the graph of CD$4^{+}$ T cell count. In addition, out of the 294 patients with available data, 79 (26.87%) patients have values lower than 4.5 × $10^{9}$/L and 15 patients have elevated values of more than 11 × $10^{9}$/L (Figure 7), and the normal range is 40–50%.

The typical range for creatinine is between 65 and 119 mg/dL. In this study, 73 patients (27.86%) out of the 262 with available data have creatinine values lower than 65 mg/dL (Figure 8).

## 4. Discussion

The present study revealed important information about the nutritional and biochemical markers and the contextual factors in HIV/AIDS patients in Bahrain. Although HIV/AIDS patients are more susceptible to malnutrition than the general population [1,28], the condition of underweight (malnutrition) in this study population was not found, since none of the participants had a BMI lower than 18.5 kg/m^2^ (underweight), and the participants’ mean BMI value was 27.20 kg/m^2^ (overweight).

It has been well established based on several studies that people living with HIV/AIDS require more energy to compensate for poor absorption, adverse drug effects, and recurrent infections [22]. Earlier research suggested that HIV/AIDS patients were generally malnourished, and that the economy and food insecurity can have deleterious impacts on patients’ nutritional status [29]. The findings of several studies revealed a higher prevalence of malnutrition among HIV/AIDS patients due to insufficient fulfillment of their basic nutritional needs [1,28,30,31,32]. On the contrary, other studies have shown that over-nutrition (overweight/obesity) is more prevalent among HIV/AIDS patients [33,34]. Similarly, the current study observed that under-nutrition was less common when compared to over-nutrition among participants. Therefore, HIV/AIDS patients in Bahrain do not lack food, and food abundance is not an issue, as none of the participants reported a concern regarding food availability or excess compared to studies conducted in other regions such as Africa [30], and rural parts of Asia [35].

The analysis of patients’ dietary intake of macronutrients (total proteins, lipids, and carbohydrates) and total calories in the present study revealed that the majority of patients’ intakes were either within or above the recommended RDA levels. These results are consistent with several other studies in which HIV/AIDS patients had sufficient macronutrient intake [33,34]. However, 38.26% of the patients in the present study had insufficient total calories (32.09% among males, and 6.17% among females). A similar dietary pattern was also found in the study of Nti (2012) [30] in Ghana in which HIV/AIDS patients’ total calories were far less than what was recommended by the WHO (20% to 30% above RDA) for adults to help maintain body weight in HIV^+^ individuals [22]. Although most Ghanaian diets are carbohydrate-based, HIV^+^ individuals’ protein intakes exceeded the recommended RDA for protein. Inadequate calorie intake can result in the use of protein as a source of energy so that the body can compensate for insufficient energy [30]. The diets of participants in the current study were found to be undiversified, although sufficient to closely reach the RDA recommendations. The findings of this study reveal that there was no variety in the intake of food from various food sources such as fruits, vegetables, and higher-fiber carbohydrates such as oatmeal, wholegrain bread, or brown rice, even though the amounts of food consumed were sufficient among patients. Martín-Cañavate et al. [36] also reported that participants’ diets were not well balanced in terms of food choices, whereas Onyango et al. [37] used a diversified meal plan that contributed to resistance to illness and infections in AIDS patients. The association between weight gain, ART, and changes in BMI was investigated by several researchers [19,38,39]. They stated that the risks of muscle wasting and being undernourished in HIV^+^ patients were reduced as the ART treatment period increased. This could be attributed to the fact that ART treatment for a long duration improves immunity and reduces risk of frequent infections, diarrhea, and vomiting, resulting in improved appetite, enhanced dietary consumption, and better nutritional status. In patients who were mostly treated with ART/highly active antiretroviral therapy (HAART), their nutritional status favored a higher prevalence of overweight [39]. Therefore, the prevalence of overweight/obese categories among participants in the current study can also be related to the ART treatment protocol carried out in HIV/AIDS patients in SMC. This situation was supported by the participants’ statement that AR elevated their appetites and caused weight gain. The results of the biochemical markers in the present study show a significant positive correlation between CD$4^{+}$ T cell count and the higher BMI kg/$m^{2}$ of patients. Similarly, the study of Khatri et al. [40] shows that lower BMI is strongly correlated with the progression of AIDS, thus negatively affecting weight and compromising patients’ ability to consume food [40]. Several studies have shown that CD$4^{+}$ T cell count and albumin levels are the two main markers that can be considered the first indices to look at in terms of AIDS progression. The common association between these studies is that hypoalbuminemia, defined as albumin concentrations <34 g/L, along with a CD$4^{+}$ T cell count below 500 cells/${\mathrm{mm}}^{3}$, are associated with more rapid progression of AIDS [41,42,43,44,45]. The results of the current study were found to be consistent with the results of these studies since a positive correlation was recorded between CD$4^{+}$ T cells and albumin. Several other studies have also shown that hematological abnormalities in hemoglobin, HCT, and MCV were prevalent among HIV/AIDS patients and associated with CD$4^{+}$ T cell count [42,46,47,48]. The results of the current study also show that low hemoglobin, HCT, and MCV were associated with a lower CD$4^{+}$ T cell count.

Elevated creatinine levels along with a low CD$4^{+}$ T cell count were found to be significantly associated with renal disease in HIV-infected individuals in several studies [49,50]. Many participants in the current study had normal levels of creatinine, and a negative correlation was found between the CD$4^{+}$ T cell count and creatinine.

This study had several limitations. First, the FFQ interview was conducted in a small sample of patients. This is not representative of the full population. The patients were under different conditions and different treatments. The different stages of HIV were another limitation. All of the patients had different lifestyles and different dietary habits.

All of these limitations could impact the final outcomes of this study, since we did not include all of the possible factors that could impact HIV progression. Future studies should include all of the relevant factors, applying analytical strategies using Machine Learning Models.

## 5. Conclusions

This study concluded that nutritional status proved to be related to important nutritional and biochemical alterations associated with HIV/AIDS. None of the participants had a BMI lower than 18.5 kg/m^2^ (underweight). The analysis of patients’ dietary intake of macronutrients and total calories revealed that the majority of patients’ intakes were either within or above the recommended RDA levels. The diets of participants were found to be undiversified but sufficient to closely reach the RDA recommendations. Lower hemoglobin, HCT, albumin, and WBCs were associated with the lower CD$4^{+}$ T cell count. This association proves its strong connection to AIDS-defining illness and progression. Higher BMI was associated with lower Hb, HCT, albumin, and WBCs, whereas the decreased levels of hemoglobin and HCT point to anemia, known to be prevalent in HIV patients, and the reduction in CD$4^{+}$ T cell count indicates immunosuppression in the patients. Finally, the HIV/AIDS community is facing two types of nutritional disorders, under-nutrition and overweight/obesity, both of which are associated with AIDS progression.

## Figures and Tables

**Table 1:** Selected demographic characteristic of the study population.

Patients’ Characteristics	N %
**Age range (year)**	60–85
**Gender**	
**Male**	248 (80.51%)
**Female**	52 (16.88%)
**Nationality**	
**Bahraini**	308
**Education**	
**Primary**	14 (17.28%)
**Intermediate**	13 (16.04%)
**Secondary**	32 (39.50%)
**Diploma/B.Sc.**	21 (25.92%)
**Higher education**	1 (1.23%)

**Table 2:** Daily consumption of selected foods from carbohydrates, proteins, caffeinated drinks, vegetables, and fruits.

Selected Food	Count (n)	% Responses
Carbohydrates		
White bread	73	90.12%
White rice	64	79.01%
Potatoes	15	18.51%
Protein		
Eggs	45	55.55%
Yogurt	31	38.27%
Beans	6	7.40%
Caffeinated drinks		
Tea	75	92.59%
Coffee	56	69.13%
Soft drinks	5	6.17%
Vegetables		
Arugula (rocket)	45	55.55%
Onions	39	48.14%
Tomatoes	25	30.86%
Fruits		
Apples	51	62.96%
Oranges	32	39.50%

**Table 3:** Macronutrient and calorie adequacy of participants’ diets based on FFQ.

Nutrients	* RDA Recommendations	Mean ± SD	% Below Recommended RDA Intake	% Within Recommended RDA Intake	% Above Recommended RDA Intake
Total calories (kcal/day)	Male: 2200–2420 Female: 1760–2200	2232 ± 509.96 2290 ± 425.46	32.09 6.17	9.89 2.47	28.39 20.99
Total proteins (g/day)	Male: 63–65 Female: 52–53	66.76 ± 36.36 65.35 ± 11.70	2.47 6.17	27.16 12.35	40.74 11.11
Total lipids (g/day)	Male: 49–54 Female: 39–49	36.36 ± 10.92 37.04 ± 7.74	9.87 8.64	2.47 1.23	58.03 19.76
Total carbohydrates (g/day)	Male: 250–300 Female: 190–250	364.04 ± 73.46 333.18 ± 87.68	0.00 0.00	0.00 0.00	70.37 29.63

FFQ: food frequency questionnaire, RDA: recommended daily allowance, range [22,23] *.

**Table 4:** Description of subjects’ BMI classification.

	* BMI Classification				
	Normal (*n*) 18.5–24.9	Overweight (*n*) 25–29.9	Obese Class I (*n*) 30–34.9	Obese Class II (*n*) 35–39.9	Obese Class III (*n*) ≥40
Classification *n* of participants	31	30	15	4	1
%	38.27	37.03	18.51	4.93	1.23

BMI = body mass index * [25].

**Table 5:** Two-tailed Pearson correlation between BMI and macronutrient content of diet.

		BMI kg/m^2^	Total Calories (kcal/day)	Total Carbohydrates (g/dL)	Total Lipids (g/dL)
Total calories (kcal/day)	Correlation	0.740 *			
	*p*-value	0.000			
Total carbohydrates (g/dL)	Correlation	0.744 *	0.907 *		
	*p*-value	0.000	0.000		
Total lipids (g/dL)	Correlation	0.701 *	0.862 *	0.867 *	
	*p*-value	0.000	0.000	0.000	
Total proteins (g/day)	Correlation	0.715 *	0.814 *	0.746 *	0.695 *
	*p*-value	0.000	0.000	0.000	0.000

* Correlation is significant at *p* < 0.01.

**Table 6:** The mean ± standard deviation of the chosen markers.

Markers	* Reference Range	Mean ± SD
CD$4^{+}$ T cell count	(500–1200 cells/${\mathrm{mm}}^{3}$)	620.91 ± 673.40
Albumin	(35–53 g/L)	42.77 ± 5.62
Hb	Male: (14–18 g/L)Female: (12–16 g/L)	13.51 ± 2.31
HCT	40–50%	42.15 ± 7.03
MCV	80–100 fl	83.13 ± 10.28
WBCs	4.5–11$\times 10^{9}$/L	6.16 ± 2.59
Creatinine	65–119 mg/dL	84.19 ± 57.28

CD$4^{+}$ T cell count: cluster of differentiation CD4^+^ T helper cell count, Hb: hemoglobin, HCT: hematocrit, MCV: mean corpuscular volume, WBCs: white blood cells * [26,27].

**Table 7:** Two-tailed Pearson correlation between nutritional and biochemical markers.

		Albumin	Cr	Hb	HCT	WBCs	MCV
Creatinine	Correlation coefficient	−0.136					
	*p*-value	0.069					
Hb	Correlation coefficient	0.619 **	0.104				
	*p*-value	0	0.148				
HCT	Correlation coefficient	0.612 **	0.081	0.933 **			
	*p*-value	0	0.255	0			
WBCs	Correlation coefficient	−0.016	0.068	−0.026	−0.008		
	*p*-value	0.821	0.342	0.706	0.911		
MCV	Correlation coefficient	0.091	0.046	0.374 **	0.349 **	0.071	
	*p*-value	0.2	0.52	0	0	0.293	
CD4^+^ T cells	Correlation coefficient	0.271 **	−0.02	0.161	0.183 *	0.165 *	−0.041
	*p*-value	0.002	0.817	0.053	0.027	0.047	0.627

CD$4^{+}$ T cell count: cluster of differentiation CD4^+^ T helper cell count, Hb: hemoglobin, HCT: hematocrit, MCV: mean corpuscular volume, WBCs: white blood cells, Cr: creatinine. * Correlation is significant at *p* value ˂ 0.05. ** Correlation is significant at *p* value ˂ 0.01.

**Table 8:** Regression analysis against CD$4^{+}$ T cell count.

Model	Coefficients		t	Sig.
	B	Std. Error		
(Constant)	−801.898	782.798	−1.024	0.308
Albumin	39.275	16.353	2.402	0.018
Creatinine	−0.049	1.013	−0.048	0.961
Hb	−34.312	104.535	−0.328	0.743
HCT	19.406	35.736	0.543	0.588
WBCs	46.563	27.002	1.724	0.087
MCV	−11.122	6.903	−1.611	0.110

CD$4^{+}$ T cell count: cluster of differentiation CD4^+^ T helper cell count, Hb: hemoglobin, HCT: hematocrit, MCV: mean corpuscular volume, WBCs: white blood cells.

## Data Availability

The data used in this study are available upon request.
